# Virchow’s Views on Cellular Pathology

**Published:** 1860-06

**Authors:** G. H. E. Baumgarten


*Virchow's Views on Cellular Pathology.* By G. H. E. Baumgarten, M. D.—The researches of Professor Rudolphus Virchow on "Cellular Pathology, based on Physiological and Pathological Histology," have not been accessible to a great majority of the profession, on account of the German garb in which they were clothed. In the *British and Foreign Medico-Chirurgical Review* for October 1856, his doctrines were criticised and dwelt upon analytically by one who appreciated their weakest points as well as their strongest claims to credence. We condense what Dr. Baumgarten has furnished to American readers as his views of Virchow's pathological theories. The basis of the theory of cellular pathology is the principle that a single cell or group of cells, operated upon by the same cause of disease, may be altered chemically, morphologically, and functionally, without any primary affection of the blood or the nervous system. As every individual cell is supposed to have an individual function, it is inferred that the function may become deranged, and disease result, and that each cell may become diseased alone, without pre-existing disease in its vicinity. With this groundwork for a general doctrine of cellular pathology, the views of Virchow are extended to embrace a wide field of scientific theorizing, much of which is doubtless true, but much must also be hypothetical.

After defining the distinctions between an animal cell and a vegetable cell, the importance of the intercellular substance is alluded to, and from the different relations which the cells bear to each other and to the intercellular substance, the mode in which tissues are built up is explained. The group of tissues in which cells have taken a specific development comprises those which are essential for the character of an animal, as nerve, muscle, and the vascular system. Those tissues in which the cell not only labors for its own nutriment, but also furnishes a certain quantity of substance outside of its walls, comprise the connective tissue and all that is related to, or developed from it, including areolar tissue, cartilage, mucous tissue, adipose tissue, and bone. Pathological formations may be classified on the same principles as the normal tissues just referred to, and, in this sense, heterology of pathological products does not exist. *Heterological* formations, they can be called, only so far as they have elements analogous to some physiological formation, existing on a spot where they ought not to grow, (*heterotopical*,) or at a time when they should not exist, (*heterochronical*,) or only in a greater quantity than is proper, (*heterometrical developments*.) The physiology of the nutritive process must be understood before the pathological condition can be thoroughly intelligible.

In the processes of active hyperæmia, the muscular elements of the artery are essentially active, relaxation speedily following irritation. The dilatation of the vessel depends in all cases on a sort of paralysis of the muscular coat, and "active hyperæmia," so called, consequently does not depend on any vascular action. The greater or less quantity of blood which flows through a part is not the cause of difference in nutrition. If we diminish the quantity of nutritive material, we can prevent a part from absorbing much, but it does not follow from this, that we can compel a part to assimilate more material by offering more blood te it. Nor does an increase in the nutritive processes of a part depend so much on a greater amount of blood in general or in part itself, as on certain states of the tissues, (*irritation*,) which change its affinities to the constituents of the blood, or on the presence of specific substances in the latter, which are particularly attracted by certain parts of the tissues. An element, destined to abstract substances from the blood, must be intact; any alteration of its molecular, physical, or chemical properties, by disease, will alter the power of executing this attraction. Cellular pathology merely demands that this law, so true in regard to the larger organs, should be admitted as equally applicable to the smaller organs and elements; that it be conceded that an epidermic cell, a cartilage cell, etc., also possesses in some measure the power of attracting materials from the blood according to its wants. The various distinctions between the humoro-pathological doctrine and those of the cellular pathology are pointed out, especially in the view of the blood being an independent formation, regenerating itself by its own resources. The theory of Virchow denies this, and considers the blood as constantly dependent on other organs. The history of the pathology of pyæmia, embolia, and the chemical dyscrasiæ, properly form a part of this branch of the subject, which is discussed at length in the original paper of Virchow. In his theory of independent cell-action, and in his refutation of the doctrine of the neuro-pathologist, he remarks that "every element can be excited to action without any influence or mediation of the nervous system;" and this observation is extended to the different action of different irritants on nearly all excitable parts.

In the limits of this abstract, we can, in addition to the points already considered, merely refer to the remaining subjects of Dr. Baumgarten's analysis of Virchow's views. These are, the phenomena of nutritive irritation, increased assimilation from a direct external irritant, formation, changes in the development of cells, the definition and pathology of inflammation in all its manifold phases, the difference between normal and pathological developments, the formation of mucus and pus, and a number of other materials for reflection and sober consideration. To these are added Dr. Baumgarten's recollections of other views of Virchow, as contained in his lectures and writings. The subject is so minutely histological in its details, that a good deal of the interest which might otherwise be felt in it is lost to those who may find a difficulty in comprehending the refinements of division in which the theory indulges.—(*St. Louis Medical and Surgical Journal*, January 1860.)

